# Single-cell transcriptomics identifies senescence-associated secretory phenotype (SASP) features of testicular aging in human

**DOI:** 10.18632/aging.205538

**Published:** 2024-02-12

**Authors:** Junxian He, Jindong Li, Yanqing Li, Zhenhan Xu, Menghui Ma, Haicheng Chen, Peigen Chen, Linyan Lv, Xuejun Shang, Guihua Liu

**Affiliations:** 1 Reproductive Medicine Center, The Sixth Affiliated Hospital, Sun Yat-Sen University, Guangzhou 510655, China; 2 Biomedical Innovation Center, The Sixth Affiliated Hospital, Sun Yat-Sen University, Guangzhou 510655, China; 3 Department of Andrology, Jinling Hospital, The First School of Clinical Medicine, Southern Medical University, Nanjing 210002, China; 4 Guangdong Engineering Technology Research Center of Fertility Preservation, Guangzhou 510655, China; 5 Department of Urology, Central South University Xiangya School of Medicine Affiliated Haikou Hospital, Haikou 570100, China

**Keywords:** single-cell transcriptome, senescence-associated secretory phenotype (SASP), aging testis, spermatogenesis microenvironment

## Abstract

The male reproductive system experiences degradation with age, predominantly impacting the testes. Testicular aging can result in failure to produce physiological testosterone levels, normal sperm concentrations, or both. However, we cannot predict the onset of testicular aging in advance. Using single-cell RNA sequencing (scRNA-seq) from Gene Expression Omnibus (GEO) database, we conducted cell-cell communication network of human testis between older and young group, indicating Leydig cells’ potential role in spermatogenesis microenvironment of aging testis. And we depicted the senescence-Associated Secretory Phenotype (SASP) features of aging testis by identifying differentially expressed senescence-associated secretory phenotype (SASP)-related genes between two group. Notably, *IGFBP7* mainly expressed in Leydig cells of those differentially expressed SASP-related genes in aging testis. Furthermore, *IGFBP7* protein located in the interstitial compartment of older mice confirmed by immunofluorescence and highly expressed in both human seminal plasma and mouse testis in the older group confirmed through Western blot. Together, our findings suggest that *IGFBP7* may be a new biomarker of testicular aging.

## INTRODUCTION

Testicular aging, leading to a decrease in the levels of testosterone, decline in sperm quality, a decline in fertility, dysfunction body, mental and psychological disorders. To treat the decline of testosterone caused by testicular aging, testosterone replacement therapy (TRT) has been proposed, but it has great side effects including prostate cancers, uncontrolled congestive heart failure, severe lower-urinary-tract symptoms, and erythrocytosis [1]. In addition, male infertility is a growing concern due to sharp decline of sperm concentration and total sperm count worldwide especially in the aging male [2, 3]. More importantly, several research have reported that increasing male age is significantly associated with bad reproductive outcome [4–6]. Despite aging being a universal, multifactorial, progressive, and irreversible process, there have been numerous attempts to delay or prevent aging in general. In the modern societies where paternal age is on the rise, research on testicular aging is increasing. Moreover, the age-related decline in testicular function also has a broader impact on overall health [7–9]. However, there has been limited consensus on the process of testicular aging [10, 11]. So, it is an urgency to find out marker of testicular aging.

Both germ cells and the spermatogenesis microenvironment play an important role in spermatogenesis. It has been observed that the testis of older man is more likely to have decreased in germ cell and malfunction in spermatogenesis microenvironment [12, 13]. For the spermatogenesis microenvironment, more and more studies pay attention to the role of Sertoli and Leydig cells [14, 15]. Sertoli and Leydig cells show significant abnormalities with age, such as decreased number, morphological variations, organelle aging, abnormal hormone secretion, and blood–testicular barrier defects. Cellular senescence is a cellular stress response triggered by molecular damage, such as replication failure, activation of abnormal oncogenes, or chemotherapy treatment.

The senescence-associated secretory phenotype (SASP) is known as secreted by senescent cells which have the therapeutic potential to aging [16, 17]. Senolytic is a new approach to kill senescent cells selectively, SASP-centered approaches are emerging as alternatives to target senescence-associated diseases. For now, there are few studies on SASP in aging testis. Herein, we downloaded single-cell RNA sequencing (scRNA-seq) from Gene Expression Omnibus (GEO) database. According to age stratification, patients were divided into older and young groups. And then, we analyzed the differences in testicular cell-cell communication networks between older and young groups, recognized the differential expressing SASP-related genes and provided a foundation for further exploration of treating testicular aging.

## MATERIALS AND METHODS

### Single-cell data acquisition and preprocessing

All raw data files were downloaded from Gene Expression Omnibus (GEO) database, including NCBI accession number GSE120508 (Donor_1, Donor_2, Donor_3), GSE215754 (Y1, HA1, HA2), GSE153947 (Normal_1, Normal_2, Normal_3) and GSE182786 (Young_1, Young_2, Young_3, Young_4, Older_1, Older_2, Older_3, Older_4, Older_5, Older_6, Older_7, Older_8). Participants were categorized into two groups: the older group and the young group. We employed an age-based classification, with individuals aged over 60 assigned to the older group, and those under 60 categorized as the young group. The Cell Ranger (v.7.0.1, 10x Genomics) was used to demultiplex the FASTQ reads align raw reads to the human reference genome (GRCh38, 10x Genomics), and generated the gene-cell unique molecular identifier (UMI) matrix for each sample. R package Seurat (v.4.3.0.1) was used to processes the count matrices. First, we filtered cells with high mitochondrial gene expression (>8%) by fitting the expression of mitochondrial genes to a normal distribution and applying a false discovery rate (FDR) threshold of <0.01. Second, we removed cells with a low number of genes detected, specifically those with fewer than 1000 genes. This step helped eliminate low-quality cells that may have been subject to technical artifacts. Third, we used the DoubleFinder (v. 2.0.3) to identify potential doublets and used a cutoff of the 92.5th percentile for the doublet score. Cells exceeding this threshold were considered potential doublets and were subsequently removed from the analysis. Following these quality control steps, we obtained a final dataset comprising 86, 626 single cells for the following analysis.

We used the harmony (v. 2.0.3) to integrate the individual samples and identify common sources of variation. The data were normalized by using “NormalizeData” function, ensuring that the number of UMIs in each cell was equal to the median UMI across the entire dataset. Additionally, a log-transformed was applied to the data. “FindVariableFeatures” function was used to identify the top 2,000 highly variable genes. Subsequently, the gene expression matrix was scale and center using the “ScaleData” function. For dimensionality reduction and visualization of the data, we performed principal component analysis (PCA) using the RunPCA function based on the highly variable genes. The resulting top 20 principal components were then used for uniform manifold approximation and projection (UMAP) analysis to visualize the clusters in 2D space. Finally, clustering was performed using the Leiden community detection algorithm, which allowed us to identify distinct cell populations based on their transcriptional profiles.

### Annotation of cell clusters

To assign the major cell types for each cluster, we followed a two-step approach. First, we performed differential expression analysis using “FindAllMarkers” function with default parameters. By comparing each cluster against all other clusters, we identified genes that showed significant differential expression. In the next step, we aimed to assign cell types to the clusters by examining the presence of known cell-type-specific genes among the top rank of differentially expressed genes in each cluster. By referencing established knowledge about marker genes for specific cell types, we determined the most likely cell type for each cluster based on these differentially expressed genes.

### Cell-cell communication analysis by CellCall

We conducted cell-cell communication analysis using CellCall [18] (v1.0.7). To ensure data integrity, we performed quality inspection and normalization on both the young and older datasets. Following the official workflow and default parameter settings, we loaded the young and older group datasets separately into CellCall. To identify potential ligand-receptor interactions between cell types in the young and older group datasets, developer of CellCall applied multiple databases including the NATMI [19], Cellinker [20], CellTalkDB [21], CellChat [22] and STRING v11 databases [23] to conduct human L–R interactions. By leveraging this R package, we screened for specific interactions and examined their potential significance in the context of young and older samples.

### Differentially expressed gene analysis

To evaluate the differentially expressed SASP-related genes between young and older group, first, we applied utilized the “FindMarkers” function from the Seurst package (v4.3.0.1). SASP-related genes were downloaded from AgeAnno database [24]. In the next step, we intersected the differentially expressed genes and SASP related genes.

### Animals

Testes of 6 mice (3 C57/BL6 mice of 3-month-old and 3 C57/BL6 mice of 20-month-old) were gathered in this experiment. All the mice were bought from the Laboratory Animal Center of Sun Yat-sen University. All experimental procedures involving animals were approved by the Institutional Animal Care and Use Committee of Sun Yat-sen University.

### Western blot analysis

Total testis protein was extracted using RIPA lysis buffer (CW2333, CWBIO, China) containing proteinase and phosphatase inhibitors. Western blot analysis was conducted as previously described [25]. The primary antibodies included anti-IGFBP7 (#36930, 1:1000; SAB, USA) and anti-beta Actin Antibody (AF7018, 1:5000; Affinity, USA) antibodies. Beta-ACTIN was used as the control.

### Semen collection

Human seminal plasmas were obtained from healthy donors that announced no reproductive system diseases history. All donors have signed an approval consent form.

### ELISA

Human seminal plasmas were collected and diluted 100x and *IGFBP7* level were detected through Human Insulin-like growth factor-binding protein 7 ELISA Kit (#EK4953, SAB, USA) in accordance with the manufacturer’s instructions.

### Statistical analysis

Statistical analysis was performed using the R software (v.4.2.2). For all variables, we applied one-tailed Wilcoxon rank-sum test to assess the differences between groups. A significance threshold of *p* < 0.05 was used to determine statistical significance. Results that met this criterion were considered to have a statistically significant difference.

### Data availability

The RNA-seq matrix data used in this study were available in NCBI with accession number GSE120508 (Donor_1, Donor_2, Donor_3), GSE215754 (Y1, HA1, HA2), GSE153947 (Normal_1, Normal_2, Normal_3) and GSE182786 (Young_1, Young_2, Young_3, Young_4, Older_1, Older_2, Older_3, Older_4, Older_5, Older_6, Older_7, Older_8).

## RESULTS

### scRNA-seq analysis of human testis samples

**Figure 1:**
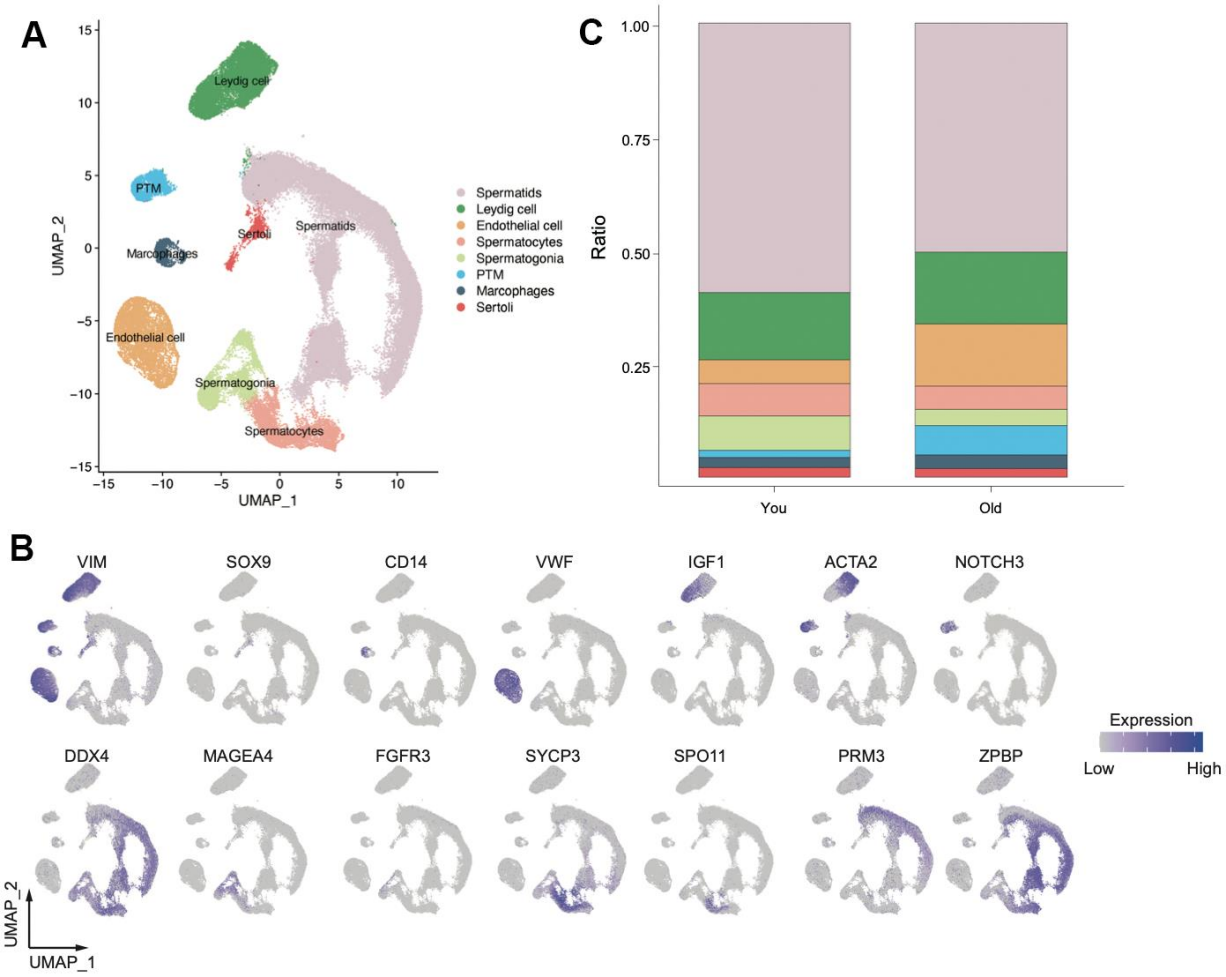
**Overview of scRNA-seq transcriptomic profiles from young and old samples (You n=9, Old n=14).** (**A**) Uniform manifold approximation and projection (UMAP) plot showing eight main clusters based on their expression of known markers (**B**). (**C**) Bar plot showing the percentage of major cell types for two group.

We downloaded FASTQ files from GEO database. A total of 23 individuals scRNA-seq was obtained, including eight individuals in older group and 14 individuals in young group (Supplementary Table 1). After quality control, we obtained a total of 86, 626 high-quality single cells by scRNA-seq (Figure 1A). Based on the known markers (Figure 1A and Supplementary Figure 1A), we identified eight main clusters: Leydig cell, Sertoli cell, macrophage, peritubular myoid cells (PTM), endothelial cells, spermatogonia, spermatocyte, spermatid. Not surprisingly, germ cells significantly decreased in older samples, especially the spermatogonia. As for somatic cell, the number of PTM and endothelial cells increased remarkable in older group. No significant difference was observed in Macrophages, Leydig cell and Sertoli cell between older and young groups (Figure 1B and Supplementary Figure 1B, 1C). Together, these data indicated that we established a comprehensive single-cell transcriptomic of human testis, tissue-structure associated cell types inferred by scRNA-seq provided a proxy for dissecting molecular changes of tissue architecture of aging testis.

### Leydig cells highly expressed differentially expressed SASP-related gene in aging testis

**Figure 2:**
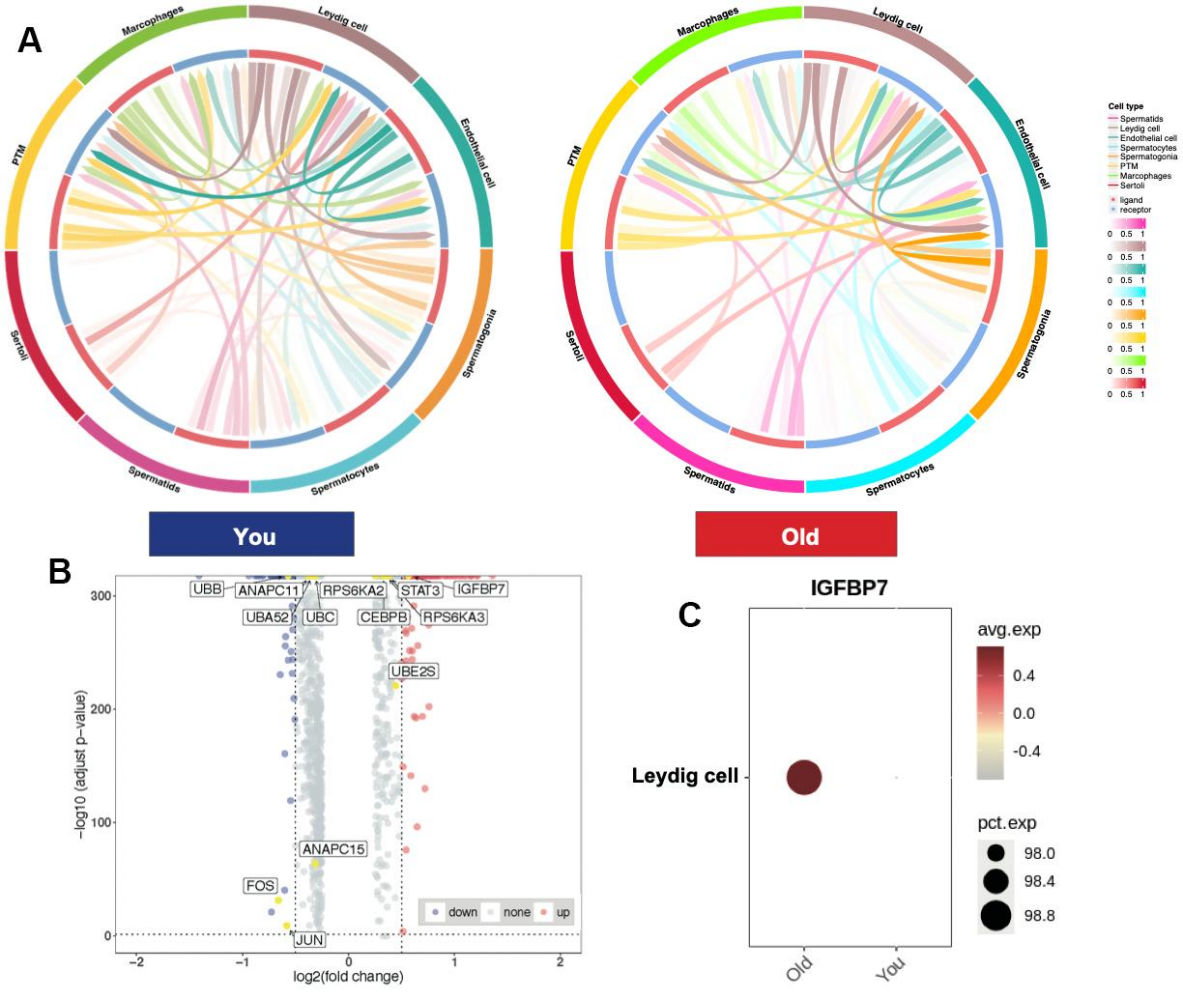
**Leydig cells highly expressed differentially expressed SASP-related gene in aging testis.** (**A**) Cell-cell communication between You and Old samples. (**B**) Volcano plot showing DEGs between You and Old samples, (Wilcoxon, adjusted *p*-values < 0.05, LogFC > 0.5) (SASP-related genes, yellow). (**C**) Dot plot showing *IGFBP7* expression enriched in older samples.

To further understand the characteristics of spermatogenesis microenvironment in older samples, we conducted cell-cell communication analysis using CellCall (v1.0.7). We found that the ligand-receptor pairs between Leydig cells and other germ cell were reduced in older group (Figure 2A), suggesting that the number of Leydig cells did not change significantly, but they did change functionally. To find out whether SASP plays a role in aging testis, we conducted differentially expressed gene (DEG) analysis between older and young group and labeled the SASP-related genes [24] in volcano plot (Figure 2B). We identified *IGFBP7* expression enriched and *JUN*, *FOS* and *UBB* were downregulated in older samples (Supplementary Table 2). Intriguingly, *IGFBP7* was highly expressed in Leydig cells, indicating that Leydig cells is a key SASP-spreading cell and may contribute to aging testis in the spermatogenesis microenvironment.

### *IGFBP7* protein highly expressed in older samples

**Figure 3:**
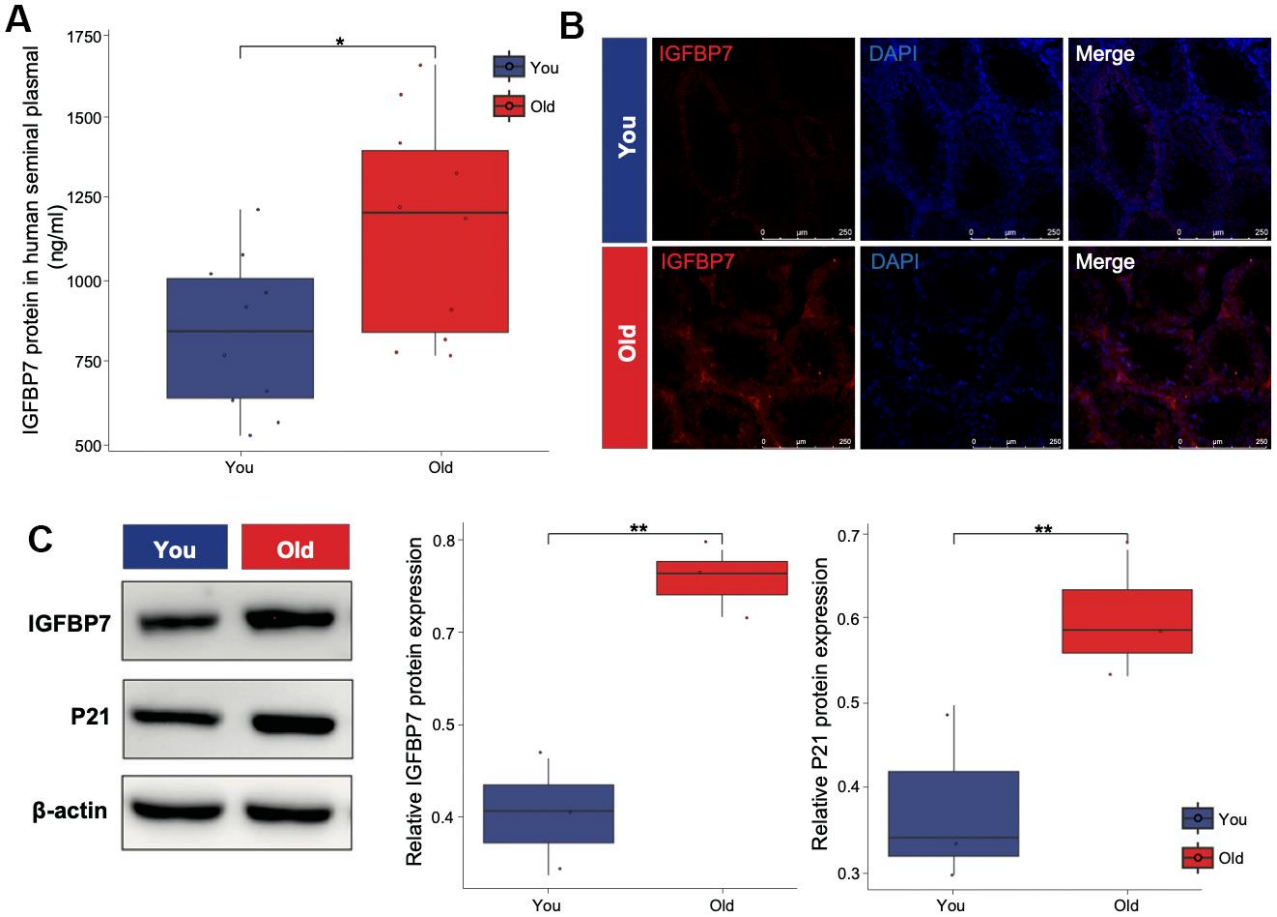
***IGFBP7* protein expression of human seminal plasma and mice testis.** (**A**) ELISA was applied to detect the expression of *IGFBP7* in human seminal plasma. (**B**) Immunofluorescence showed the localization of *IGFBP7* protein in the interstitial compartment of older mice. Scale bar: 250 μm (**C**) Western blot was applied to detect the expression of *IGFBP7* and the senescence marker P21 in testis of mice. Data depict the mean ± SD; *P < 0.05. **P < 0.01.

Senescent cells produce a series of profibrotic and proinflammatory factors, these factors are also known as SASP. *IGFBP7* are important transcriptional inducers of the SASP. *IGFBP7*, derived from senescent cells, plays a crucial role in inducing senescence in young mesenchymal stem cells. To verify whether SASP such as *IGFBP7* are secreted by Leydig cells and travel cross the seminal tubular basement membrane, we collected human seminal plasma from healthy donors and measured *IGFBP7* protein levels. By using ELISA, we found that *IGFBP7* protein could be detected in seminal plasma, and interestingly, seminal plasma *IGFBP7* expression levels were higher in the older group than in the young group (Figure 3A). We also measured the location by immunofluorescence and expression level of *IGFBP7* in the testes of C57/BL6 mice by Western blot. We found that *IGFBP7* was mainly expressed in testicular interstitial compartment, and the level of *IGFBP7* in the testes of mice in the older group was higher than that in the young group (Figure 3B, 3C), indicating that *IGFBP7* may be a new biomarker of testicular aging.

## DISCUSSION

In this study, we established a comprehensive single-cell transcriptomic of aging testis in human. By conducting the cell-cell communication network with young and older samples, we were able to identify Leydig cells’ potential role in aging testis. The ligand-receptor pairs between Leydig cells and other germ cell were significantly reduced in older samples. In line with that, multiple studies also reported that Leydig cells from older men display malfunction, including lower production of testosterone [13, 26]. So, application of TRT to treat male infertility was mentioned [27]. However, the concern of promoting prostate cancer limits the use of TRT [28]. And the sole unequivocal indication for TRT is as replacement therapy for men with pathological hypogonadism. It does not apply to older infertile men with normal testosterone levels. There is an urging to find out other therapeutic strategies for aging men.

Nie recently generated single-cell transcriptomic sequencing and analysis in testes of young and old human exploring the correlation between testicular aging and elevated body mass index (BMI) and providing a comprehensive examination of functional changes in various cell types within aging testes [13]. However, they primarily focused on delineating functional changes in various cell types within the aging testes, with a notable absence of emphasis on senescence-associated secretory phenotype (SASP) factors in the context of testicular aging.

Cellular senescence promotes tissue remodeling through three sequential processes: a stable proliferative arrest; a secretory phenotype (SASP) that recruits immune cells and modifies the extracellular matrix; and the mobilization of nearby progenitors that repopulate the tissue [29, 30]. Senescent cells produce a series of profibrotic and proinflammatory factors, including interleukin 6 (*IL-6*), plasminogen activator inhibitor-1 (*PAI-1*), and transforming growth factor beta (*TGF-β)*, these factors are also known as SASP [31]. SASP can have both beneficial effects and detrimental consequences. The SASP mediates the tumor suppressor functions of senescence [32]. On the other hand, aging cells can develop a characteristic pathogenic SASP that drives secondary senescence and disrupts tissue homeostasis, resulting in loss of tissue repair and regeneration [33, 34]. Identifying the key SASP factors in aging testis and developing a targeted drug to eliminate them may be a potential treatment for aging men [35, 36]. Our study identifies differentially expressed SASP-related genes between young and older human testis samples. Compared with young human testis, we identified upregulated SASP-related genes *IGFBP7* and downregulated SASP-related gene including *JUN*, *FOS* and *UBB*. *IGFBP7* are important transcriptional inducers of the SASP [37]. *IGFBP7* derived from senescent cell has been proved to be key components that triggers senescence in young mesenchymal stem cells [38]. *IGFBP7* mainly expressed by Leydig cells from older men. And we confirmed the *IGFBP7* protein both in human seminal plasma and mice testis sample. Named by GFBP-related proteins, insulin-like growth factor-binding protein 7 (IGFBP7) is one of the proteins belonging to the *IGFBP* superfamily. *IGFBP7* is a high-affinity insulin-binding protein, acting an inhibitory role by hindering proliferation and inducing apoptosis and senescence [39]. Wajapeyee and et al. reported melanocytes secreted *IGFBP7*, and it acted through an autocrine/paracrine pathway to induce senescence [40]. Previous studies have also identified dilp-binding protein ImpL2, a drosophila homolog of *IGFBP7* blocked InR activation in stem cells of drosophila testis, inducing curb of somatic cell differentiation by downregulating of PI3K/Tor signaling [41]. Higher concentrations of *IGFBP7* also indicate increasing risk of cardiovascular events [42]. These studies suggest that *IGFBP7* has the potential to serve as a key factor in testicular aging and has significant drug development value.

Removal of senescent cells increases healthy life span in murine models [43, 44]. Due to the potential of reducing the burden of senescent cells to prolong healthy lifespan and delay the onset of age-related diseases, there is increasing interest in developing sensory therapies that integrate multidisciplinary technologies such as biology, chemistry, nanotechnology, and immunology [45–49]. Senolytic tending to selectively deliminate senescent cell is a new therapeutic strategy to manage testicular aging. Multiple basic research have laid the foundation for human clinical trials [50–54]. For now, many senolytic strategies have been investigated, such as inhibitors of the antiapoptotic *BCL-2* family proteins, *HSP90* inhibitors, *USP7* inhibitors, *p53* modulators, Na/K-ATPase inhibitors. In our previous study we discovered FOXO4-DRI, a specific *FOXO4* blocker, selectively induced *p53* nuclear exclusion and apoptosis in senescent Leydig cells, which improved the testicular microenvironment and alleviated age-related testosterone secretion insufficiency [25]. Gypenoside XLIX, a type of dammarane-type saponins that have diverse biological properties, including anti-inflammatory, antithrombotic, anticancer, hepatoprotection, and neuroprotective effects, markedly suppressed the levels of *IGFBP7* and reduced the binding of *IGFBP7* to *IGF1* receptor in an acute kidney injury model, and showed great potential in releasing senescence. However, there were two limitations in our study. First, our conclusion was generated using retrospective data from public databases. Therefore, it should be validated in more prospective and multi-center aging cohorts in the future. Second, the underlying molecular mechanism of *IGFBP7* still needs to be further explored.

## CONCLUSIONS

In summary, here we provide new aspect concerning SASP factors in aging testis. Although there is limited understanding of the detailed molecular mechanisms so far, more investigation needs to be done. Collectively, our data implicate *IGFBP7* as a promising aging suppressor protein in aging testis.

## Supplementary Material

Supplementary Figure 1

Supplementary Table 1

Supplementary Table 2
