# Supplementary material for: Single-cell transcriptomics identifies senescence-associated secretory phenotype (SASP) features of testicular aging in human
Source: Aging (Albany NY). 2024 Feb 12;16(4):3350–62. doi: 10.18632/aging.205538 (PMC10929807; doi:10.18632/aging.205538)
Supplement: Supplementary Figure 1 [file aging-16-205538-s001.pdf]

# SUPPLEMENTARY FIGURE

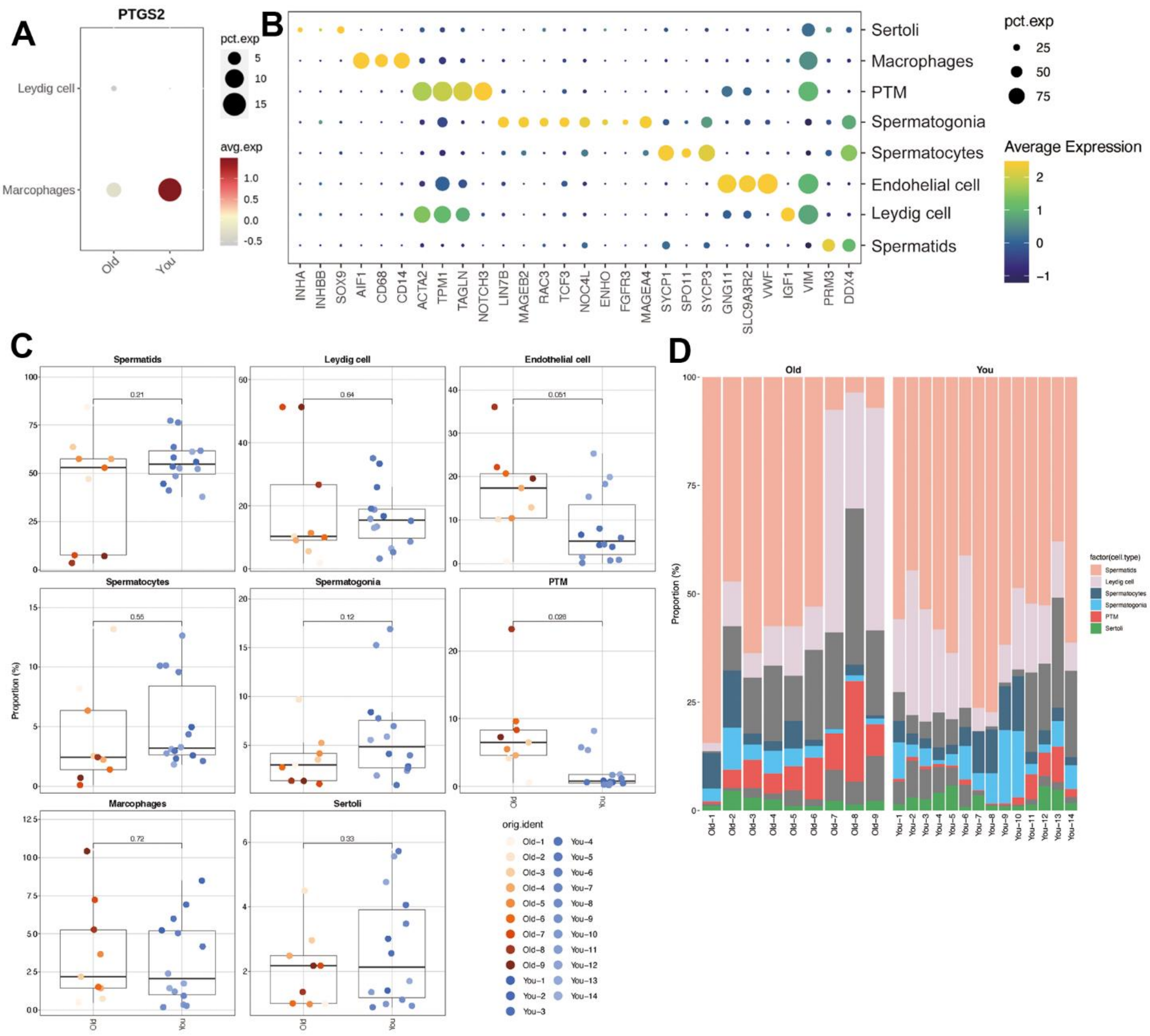


**Supplementary Figure 1.** (**A**) Expression of the senescence marker PTGS2 in the old and young groups. (**B**) Marker genes of eight main clusters. (**C**) Proportion of eight main cell types in the old and young groups. (**D**) Bar plot showing the percentage of major cell types for each sample.
