# Supplementary material for: Single-cell transcriptomics identifies senescence-associated secretory phenotype (SASP) features of testicular aging in human
Source: Aging (Albany NY). 2024 Feb 12;16(4):3350–62. doi: 10.18632/aging.205538 (PMC10929807; doi:10.18632/aging.205538)
Supplement: Supplementary Table 1 [file aging-16-205538-s002.pdf]

SUPPLEMENTARY TABLES

Supplementary Table 1. Patient information.

Name	Age	Data	Old_name
		**Young**	
Young_1	17	GSE120508	Donor_1_rep2
Young_2	24	GSE120508	Donor_2_rep1
Young_3	24	GSE120508	Donor_2_rep2
Young_4	25	GSE120508	Donor_3_rep1
Young_5	25	GSE120508	Donor_3_rep2
Young_6	26	GSE215754	Y1
Young_7	56	GSE215754	HA1
Young_8	31	GSE153947	Normal_1
Young_9	33	GSE153947	Normal_2
Young_10	55	GSE153947	Normal_3
Young_11	17-22	GSE182786	Young_1
Young_12	17-22	GSE182786	Young_2
Young_13	17-22	GSE182786	Young_3
Young_14	17-22	GSE182786	Young_4
		**Old**	
Old_1	67	GSE215754	HA2
Old_2	>60	GSE182786	Older_1
Old_3	>60	GSE182786	Older_2
Old_4	>60	GSE182786	Older_3
Old_5	>60	GSE182786	Older_4
Old_6	>60	GSE182786	Older_5
Old_7	>60	GSE182786	Older_6
Old_8	>60	GSE182786	Older_7
Old_9	>60	GSE182786	Older_8
